# Evolution of etiology, presentation, management and prognostic tool in hepatocellular carcinoma

**DOI:** 10.1038/s41598-020-61028-9

**Published:** 2020-03-03

**Authors:** Shu-Yein Ho, Po-Hong Liu, Chia-Yang Hsu, Cheng-Yuan Hsia, Yi-Hsiang Huang, Hao-Jan Lei, Chien-Wei Su, Rheun-Chuan Lee, Ming-Chih Hou, Teh-Ia Huo

**Affiliations:** 1 Department of Medicine, Taipei Veterans General Hospital, Taipei, Taiwan; 2 Department of Surgery, Taipei Veterans General Hospital, Taipei, Taiwan; 3 Department of Radiology, Taipei Veterans General Hospital, Taipei, Taiwan; 4 Department of Medical Research, Taipei Veterans General Hospital, Taipei, Taiwan; 5 Faculty of Medicine, National Yang-Ming University School of Medicine, Taipei, Taiwan; 6 Institute of Clinical Medicine, National Yang-Ming University School of Medicine, Taipei, Taiwan; 7 Institute of Pharmacology, National Yang-Ming University School of Medicine, Taipei, Taiwan; 8 Department of Internal Medicine, University of Texas Southwestern Medical Center, Dallas, Texas USA; 9 Division of Gastroenterology and Hepatology, University of Michigan, Ann Arbor, MI USA

**Keywords:** Cancer, Gastrointestinal cancer, Liver cancer, Cancer, Oncology

## Abstract

Hepatocellular carcinoma (HCC) is the fourth leading cause of cancer-related death worldwide, but its current status is unclear. We aimed to investigate the evolution of etiology, presentation, management and prognostic tool in HCC over the past 12 years. A total of 3349 newly diagnosed HCC patients were enrolled and retrospectively analyzed. The comparison of survival was performed by the Kaplan-Meier method with the log-rank test. Hepatitis B and C virus infection in HCC were continuously declining over the three time periods (2004–2007, 2008–2011, 2012–2015; p < 0.001). At diagnosis, single tumor detection rate increased to 73% (p < 0.001), whereas vascular invasion gradually decreased to 20% in 2012–2015 (p < 0.001). Early stage HCC gradually increased from 2004–2007 to 2012–2015 (p < 0.001). The probability of patients receiving curative treatment and long-term survival increased from 2004–2007 to 2012–2015 (p < 0.001). The Cancer of Liver Italian Program (CLIP) and Taipei Integrated Scoring (TIS) system were two more accurate staging systems among all. In conclusion, the clinical presentations of HCC have significantly changed over the past 12 years. Hepatitis B and C virus-associated HCC became less common, and more patients were diagnosed at early cancer stage. Patient survival increased due to early cancer detection that results in increased probability to undergo curative therapies.

## Introduction

Hepatocellular carcinoma (HCC) is the most common primary liver cancer and the fourth leading cause of cancer-associated mortality in 2018 globally^1^. The incidence of HCC is high in East Asia with male prediomance^2–4^. Also, HCC is the second most common reason for cancer-related mortality in Taiwan^5^, an endemic area for hepatitis B virus (HBV).

HBV infection in Taiwan in mainly due to perinatal mother-to-infant transmission of the virus. The carrier rate of hepatitis B surface antigen (HBsAg) in general population was as high as 15–20%^6^. Prospective cohort and meta-analyses studies showed a 10- to 100-fold increase in the risk of HCC development among subjects chronically infected with HBV^2,7^. In Taiwan, the vaccination program against HBV was initiated in July 1984, and has reduced the incidence of HCC in children and adolescent successfully^8,9^. In addition to HBsAg, positive serum HBV e antigen and HBV DNA level were also associated with increased cancer risk^10^. Cumulative evidence showed that antiviral therapy might suppress HBV replication and lead to reduced risk of HCC formation^11–13^. HCV infection is another risk factor for HCC. The risk for HCC development in patients with serologically confirmed HCV infection was estimated to be 17-fold^2^. Notably, in recent years, nonalcoholic fatty liver disease (NAFLD), which is often associated with obesity and diabetes mellitus, emerged as potentially new risk factor for HCC^3,14,15^.

For patients with early stage HCC, surgical resection, liver transplantation or local ablation therapy are usually indicated, with 5-year survival rate up to 75%^14,16^. Transarterial chemoembolization is often considered for unresectable HCC without liver decompensation, and 2-year survival rate was estimated 20–25%^3^. Alternatively, in patients with far-advanced or terminal stage HCC, a dismal prognosis is anticipated and usually best supportive care could be provided^3,4^. Due to the fast changes in cancer prevention and treatment selection over the past decades, the current disease pattern and therapeutic strategy for HCC are largely unknown. We aimed to investigate the evolution of etiology, presentation, management, survival and prognostic tool in HCC patients from 2004 to 2015.

## Patients and Methods

### Patients

Over a 12-year period from 2004 to 2015, 3349 patients with newly diagnosed HCC in Taipei Veterans General Hospital, Taiwan, were prospectively identified and retrospectively analyzed. This study was categorized into three time periods by each 4-year interval, 2004–2007, 2008–2011 and 2012–2015, for comparison. Part of the enrolled patients have been described in our previous studies^16,17^. The baseline demographics, etiology of liver disease, serum biochemistry, tumor burden, performance status, cancer stage and treatment modality were comprehensively collected. All these patients were staged according to Barcelona Clínic of Liver Cancer (BCLC) staging at the time of diagnosis. The survival of patients was inspected and calculated every 3–4 months until death or dropout from the follow-up program and was cross-referenced from the database of Taiwan National Cancer Registry. This study was approved by the Institutional Review Board (IRB) of Taipei Veterans General Hospital, Taiwan, and complies with the standards of Declaration of Helsinki and current ethical guidelines. Waiver of consent was approved by the IRB of Taipei Veterans General Hospital, and patient’s personal information was anonymized and de-identified prior to analysis.

### Diagnosis and definitions

The diagnosis of HCC was based on the findings of typical radiological features contrast-enhanced dynamic computed tomography (CT) and magnetic resonance imaging (MRI) or histology confirmed if atypical radiological features^3,14^. The performance status was evaluated by using Eastern Cooperative Oncology Group (ECOG) scale: 0 (asymptomatic) to 4 (confined to bed)^18^. All clinical data analyzed in this study were recorded at the time of diagnosis.

### Surveillance

Current practice guidelines from the American Association for the Study of Liver Diseases (AASLD), European Association of the Study of Liver Diseases (EASL) and Asia-Pacific Association of the Study of Liver diseases (APASL) recommend surveillance for patients at high risk for hepatocellular carcinoma^2,3,5^. The combined use of serum ɑ-fetoprotein (AFP) level and abdominal sonography was regularly performed every 4–6 months for screening high-risk subjects including chronic hepatitis B and C, and subclinical or overt cirrhosis^2,19^.

### Treatment

The newly diagnosed HCC patients at Taipei Veterans General Hospital were discussed in the multidisciplinary board for diagnosis confirmation and treatment recommendation. The criteria of surgical resection for HCC were (1) patients with tumor involving no more than three Healey’s segments, (2) Child-Turcotte-Pugh (CTP) class A with less than 25% of retention of indocyanine green 15 min after injection, and (3) no main portal trunk invasion or distant metastasis^20^. Radiofrequency ablation (RFA) was indicated in patients who had preserved liver function but were not eligible for surgical resection^17,21^. Transarterial chemoembolization (TACE) was performed in patients who had unresectable lesions or unwilling to receive curative treatment in the absence of distant metastasis and hepatic decompensation^14^. Systemic therapy or targeted therapy was given to selected patients with adequate liver functional reserve^3,14^. For patients with poor liver functional reserve or decreased performance status, best supportive care was given. Shared-decisions were made between physicians and patients prior to initiation of any definite treatment. In this study, resection, ablation and liver transplantation were classified as curative treatments. Other managements were collectively labeled as non-curative treatments.

### Cancer staging

According to the AASLD and EASL HCC management guidelines, the Barcelona Clínic of Liver Cancer (BCLC) is endorsed as the standard staging system for HCC^3,14^. In addition, the prognostic power for HCC among the Cancer of the Liver Italian Program (CLIP), tumor-node-metastasis (TNM) system, Tokyo score, Japan Integrated Scoring (JIS) system, Taipei Integrated Scoring (TIS) system, and Hong Kong Liver Cancer (HKLC) staging system was also compared in this study^22–25^.

### Statistics

The Chi-squared test and two-tailed Fisher exact test were used to compare categorical data. The Mann-Whitney ranked sum test or Kruskal-Wallis test was used to compare continuous variables between different groups. Data were expressed as the mean ± standard deviation (SD) and median with interquartile range (IQR). The comparison of survival distribution was performed by the Kaplan-Meier method with the log-rank test. Corrected Akaike information criteria (AICc) was obtained to reveal how the staging system correlated with patient’s survival. The AICc was chosen over Akaike information criteria to compensate for the different number of parameters in each staging system. Homogeneity was measured by χ2 test to evaluate the differences in survival among patients in the same stage within each system^26^. The lower AIC, the more explanatory and informative the staging system is^27^. A p-value < 0.05 was considered statistically significant by 2-tailed tests. All statistical analyses were performed using IBM SPSS Statistics for Windows, Version 21.0 (IBM Corp., Armonk, NY, USA).

## Results

### Baseline characteristics and staging

A total of 3349 newly diagnosed HCC patients were consecutively identified during the study period. Their baseline characteristics and clinical information are shown in Table 1. There were 1308, 1136, and 905 HCC patients in the time periods of 2004–2007, 2008–2011 and 2012–2015, respectively. The mean age was 65 years, and the majority of patients were male in these three cohorts. Regarding the etiology of liver disease, the prevalence of HBV infection continuously declined from 43% in 2004–2007 to 39% in 2008–2011 and 36% in 2012–2015 (p < 0.001). The prevalence of HCV-related HCC was 24% in 2004–2007, and gradually decreased to 22% in 2008–2011 and 20% in 2012–2015 (p < 0.001). The prevalence of cryptogenic HCC increased from 14% in 2004–2011 to 20% in 2012 to 2015 (p < 0.001). The prevalence of diabetes mellitus in HCC was 24%, 24% and 27% in 2004–2007, 2008–2011 and 2012–2015 cohort, respectively (p = 0.001).

**Table 1:** Baseline characteristics, staging and treatments.

Variables	2004–2007 (n = 1308)	2008–2011 (n = 1136)	2012–2015 (n = 905)	p
Age (years, mean ± SD)	65 ± 13	64 ± 14	65 ± 14	0.049
Male/female (%)	1009/299 (77/23)	896/240 (79/21)	659/246 (73/27)	0.005
**Etiologies of liver disease**				
HBV, n (%)	560 (43)	439 (39)	326 (36)	0.001
HCV, n (%)	315 (24)	296 (22)	178 (20)	0.001
HBV + HCV, n (%)	55 (4)	25 (2)	35 (4)	0.002
Alcohol, n (%)	186 (15)	254 (23)	185 (20)	0.001
Cryptogenic, n (%)	192 (14)	157 (14)	181 (20)	0.001
Performance status (0/1/2–4), (%)	865/158/285 (66/12/22)	537/269/332 (47/24/29)	525/310/70 (58/34/8)	0.001
**Laboratory values (mean ± SD)**				
Albumin (g/L)	3.7 ± 0.6	3.6 ± 0.6	3.6 ± 0.6	0.024
Bilirubin (mg/dL)	1.5 ± 2.7	1.0 ± 3.0	1.5 ± 2.9	0.629
ALT (IU/L)	74 ± 90	72 ± 110	63 ± 79	0.003
AST (IU/L)	102 ± 190	110 ± 290	81 ± 107	0.022
Creatinine (mg/dL)	1.2 ± 1.0	1.1 ± 1.0	1.2 ± 1.1	0.206
Sodium (mmol/L)	139 ± 4	138 ± 4.0	139 ± 4	0.395
INR of PT	1.08 ± 0.17	1.10 ± 0.19	1.12 ± 0.17	0.001
Platelet (1000 ul/L)	173 ± 100	172 ± 97	163 ± 93	0.019
AFP (ng/mL)	20518 ± 156897	25442 ± 190694	19655 ± 122192	0.886
AFP (ng/mL), median [IQR]	46 [10–770]	56 [10–1037]	31 [6–595]	
Tumor nodules (single/multiple), n (%)	772/536 (59/41)	689/447 (61/39)	661/244 (73/27)	0.001
Maximal tumor diameter (cm, mean ± SD)	5.9 ± 4.3	6.4 ± 4.6	5.8 ± 4.5	0.921
Vascular invasion (no/yes)	980/328 (75/25)	812/324 (72/28)	724/181 (80/20)	0.001
Metastasis (no/yes)	1186/122 (91/9)	1014/122 (89/11)	818/87 (90/10)	0.481
Ascites, n (%)	251 (19)	365 (32)	163 (18)	0.001
DM, n (%)	309 (24)	269 (24)	297 (27)	0.001
CTP grade (A/B/C), n (%)	963/280/65 (74/21/5)	779/275/82 (69/24/7)	691/198/16 (76/22/2)	0.001
CTP score (mean ± SD)	6.0 ± 1.6	6.3 ± 1.7	5.9 ± 1.3	0.017
**Cancer stages**				
BCLC (0/A/B/C/D), n (%)	115/338/267/394/194 (8/25/20/30/15)	83/190/142/548/173 (7/17/13/48/15)	77/258/118/416/36 (9/28/13/46/4)	0.001
HKLC (I/II/III/IV/V), n (%)	453/324/129/104/298 (35/25/10/8/22)	278/291/115/109/343 (25/26/10/9/30)	326/296/103/96/84 (36/33/11/11/9)	0.001
CLIP (0/1/2/3/4/5/6), n (%)	371/380/216/153/109/66/13 (28/29/17/12/8/5/1)	315/285/186/135/131/66/18 (28/25/16/12/11/6/2)	352/226/114/100/78/32/3 (39/25/12/11/8/4/1)	0.001
TIS (0/1/2/3/4/5/6), n (%)	462/311/157/157/128/80/13 (35/24/12/12/10/6/1)	355/229/160/151/146/74/21 (31/20/14/13/13/7/2)	368/196/100/86/101/52/2 (40/21/11/10/11/6/1)	0.001
JIS (0/1/2/3/4/5), n (%)	94/384/428/243132/27 (7/29/33/19/10/2)	111/297/330/234/134/30 (10/26/29/21/12/2)	95/384/245/124/54/ 3 (11/41/27/14/6/1)	0.001
Tokyo (0/1/2/3/4/5/6/7/8), n (%)	81/271/336/253/163/115/51/32/6 (6/21/26/19/12/9/4/2/1)	71/225/289/209/156/108/50/22/6 (6/20/25/18/14/10/4/2/1)	62/220/240/173/106/55/27/14/7 (7/24/26/19/12/6/3/1)	0.154
TNM (I//II/III/IV), n (%)	483/230/499/96 (37/18/38/7)	399/187/485/65 (35/17/43/5)	300/314/243/48 (33/35/26/5)	0.001
**Treatment modality, n (%)**				
Resection	336 (25)	272 (24)	334 (37)	0.001
Local ablation	247 (18)	228 (20)	152 (17)	0.001
Liver transplantation	5 (1)	4 (1)	6 (1)	0.819
TACE	399 (31)	336 (30)	198 (22)	0.001
Targeted therapy	2 (1)	38 (3)	75 (8)	0.001
Chemotherapy	87(6)	37 (3)	8 (1)	0.001
Radiotherapy	7 (1)	21 (2)	31 (3)	0.001
Best supportive care	225 (17)	200 (17)	101 (11)	0.001

AFP, serum alpha-fetoprotein; ALT, alanine aminotransferase; AST, aspartate aminotransferase; BCLC, Barcelona Clínic of Liver Cancer; CLIP, Cancer of the Liver Italian Program; CTP, Child-Turcotte-Pugh; DM, diabetes mellitus; HBV, hepatitis B virus; HCV, hepatitis C virus, HKLC, Hong Kong Liver Cancer; INR of PT, international normalized ratio of prothrombin time; JIS, Japan Integrated Scoring; TACE, transarterial chemoembolization; TNM, tumor-node-metastasis; TIS, Taipei Integrated Scoring.

Single tumor detection rate was 59% in 2004–2007, and the rate increased to 61% in 2008–2011 and 73% in 2012–2015 (p < 0.001). Patients with a poor performance status (status 2–4) at the time of diagnosis decreased from 22% in 2004–2007 to 8% in 2012–2015 (p < 0.001). Patients of the 2012–2015 cohort had significantly better performance status, less multiple tumor nodules, less vascular invasion, better liver functional reserve and increased overall survival when specifically compared with 2004–2007 cohort (all p < 0.001).

The proportion of patients diagnosed at early cancer stage gradually increased, whereas the proportion of terminal stage decreased in three time periods according to the BCLC, HKLC, CLIP, TIS, JIS and TNM staging systems (all p < 0.01), except Tokyo score (p = 0.154).

### Treatment

**Figure 1:**
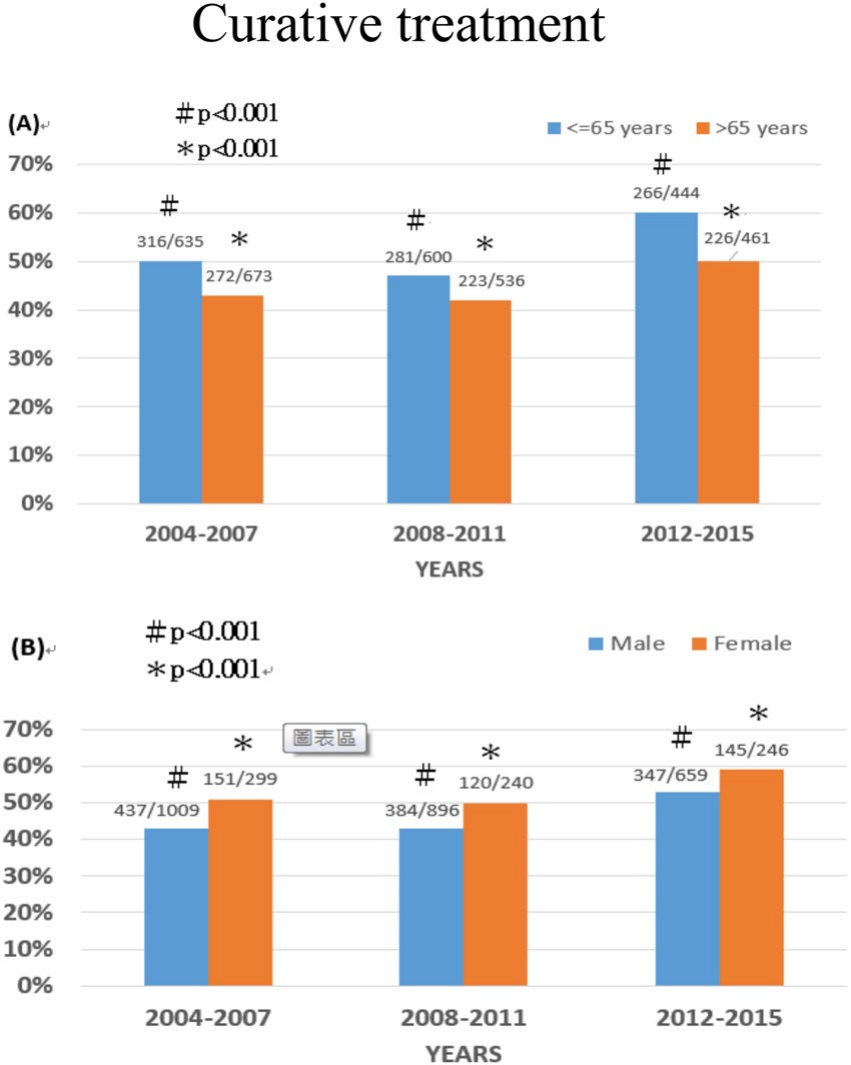
Comparison of the prevalence of patients with hepatocellular carcinoma with age ≤ 65 years vs > 65 years in 2004–2007, 2008–2011 and 2012–2015 (panel A; p < 0.001), and in males and females in 2004–2007, 2008–2011 and 2012–2015 (panel B; p < 0.001).

The probability for patients receiving curative treatment was 44% in 2004–2007 and the rate increased to 45% in 2008–2011 and 55% in 2012–2015 (p < 0.001) independent of age and sex (Fig. 1), and the rate was the highest in the 2012–2015 when compared with 2004–2007 cohort (p < 0.001). By contrast, the percentages of TACE and best supportive care consistently decreased over the three time periods (Table 1, p < 0.001).

### Prognostic performance of the seven staging systems

The prognostic accuracy of 7 HCC staging systems was evaluated in three time periods (Table 2). In both 2004–2007 and 2008–2011, the CLIP staging system had the highest homogeneity and lowest AICc, suggesting superior prognostic capability than other staging systems. By contrast, in 2012–2015, the TIS system had the highest homogeneity and lowest AICc, indicating a better prognostic performance in this time period.

**Table 2:** Prognostic performance for hepatocellular carcinoma in different staging systems.

Staging	2004–2007		2008–2011		2012–2015	
	Homogeneity (Wald χ2)	AICc	Homogeneity (Wald χ2)	AICc	Homogeneity (Wald χ2)	AICc
BCLC	459.6	13112.3	234.4	10568.5	250.1	4570.8
HKLC	508.8	13063.1	289.3	10513.6	297.4	4523.5
CLIP	594.3	12977.7	496.2	10306.8	341.5	4479.7
TIS	483.6	13088.4	425.0	10377.9	341.9	4479.0
JIS	372.4	13199.6	345.2	10457.7	222.5	4598.4
Tokyo	443.6	13128.3	387.0	10416.0	270.5	4550.4
TNM	200.4	13371.6	178.2	10624.7	120.8	4700.1

BCLC, Barcelona Clínic of Liver Cancer; CLIP, Cancer of the Liver Italian Program; HKLC, Hong Kong Liver Cancer; JIS, Japan Integrated Scoring; TNM, tumor-node-metastasis; TIS, Taipei Integrated Scoring.

### Long-term survival

**Figure 2:**
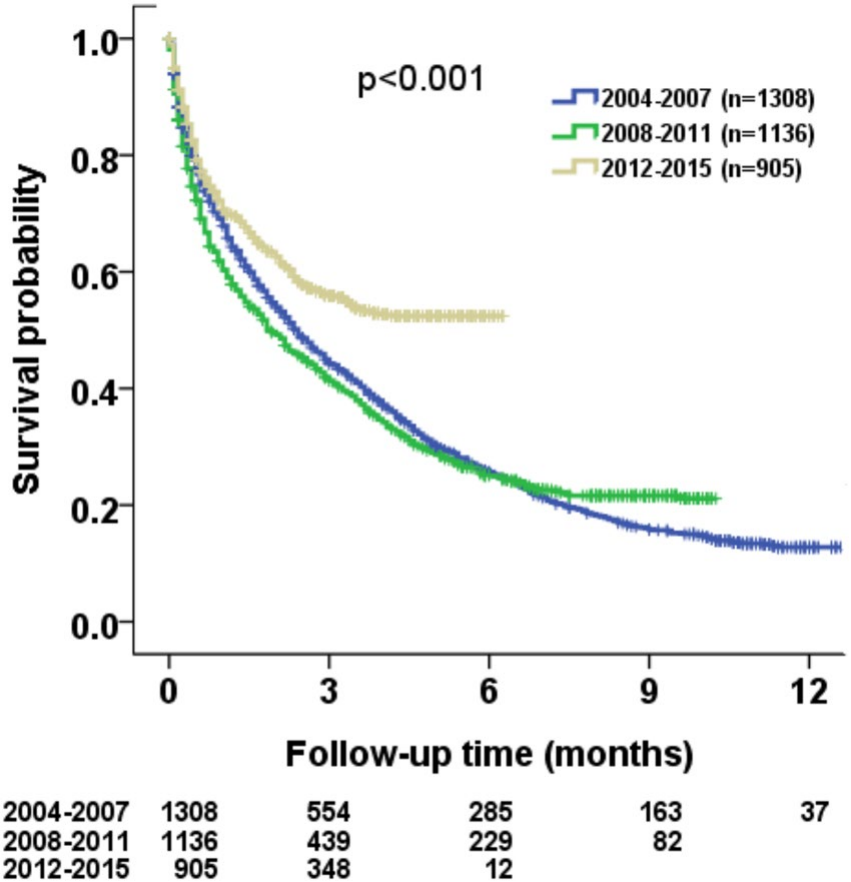
Comparison of survival distribution of all study patients in three different time periods from 2004 to 2015. Significant survival differences were found among these three cohorts (p < 0.001).

The median survival of HCC patients was 30 months (95% confidence interval [CI]): 25–32 months) in 2004–2007, 23 months (95% CI: 19–27 months) in 2008–2011, and 72 months (95% CI: not reached) in 2012–2015 cohort (Fig. 2). A total of 1051 (80%), 839 (74%) and 375 (41%) patients died in 2004–2007, 2008–2011 and 2012–2015 period, respectively. Survival analysis disclosed that patient survival tended to be better in 2012–2015 (p < 0.001). The estimated survival probability at 1, 3 and 5 years were 69%, 45%, 31% for 2004–2007 cohort, 62%, 42%, 29% for 2008–2011 cohort, and 72%, 56%, 52% for 2012–2015 cohort, respectively.

**Figure 3:**
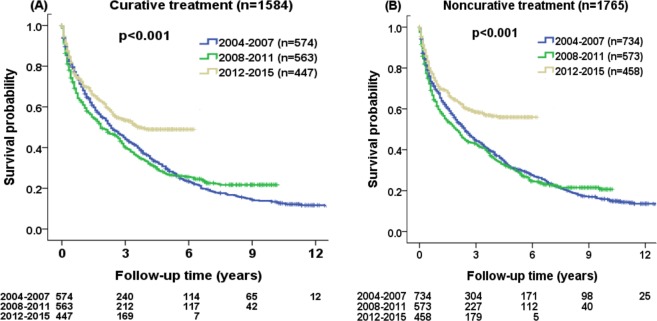
Comparison of survival distribution in three different time periods (2004–215) according to (**A**) curative treatment and (**B**) non-curative treatment. Significant differences in survival distributions were found among three different cohorts (both p < 0.001).

In subgroup analysis, the survival probability at 1, 3 and 5 years were 90%, 67%, 49% for 2004–2007 cohort, 87%, 70%, 52% for 2008–2011 cohort, and 91%, 78%, 74% for 2012–2015 cohort, respectively, in patients undergoing curative treatment. The survival probability at 1, 3 and 5 years were 53%, 26%, 15% for 2004–2007 cohort, 42%, 19%, 11% for 2008–2011 cohort, and 50%, 27%, 24% for 2012–2015 cohort, respectively, in patients undergoing non-curative treatment. There were significant survival differences among 2004–2007, 2008–2011 and 2012–2015 cohort in both treatment groups (Fig. 3A,B; both p < 0.001).

**Figure 4:**
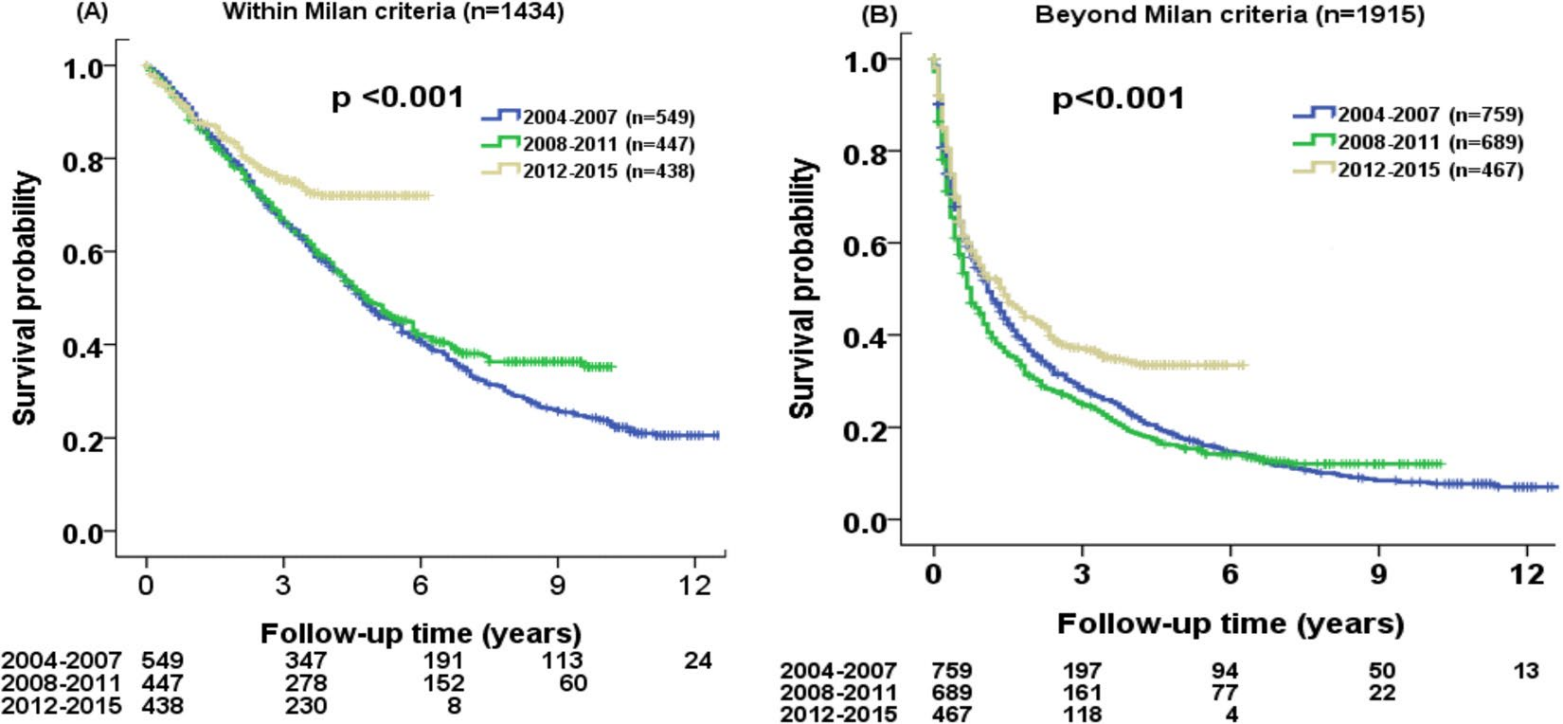
Comparison of survival distribution in three different time periods (2004–2015) according to (**A**) within Milan criteria and (**B**) beyond Milan criteria. Significant differences in survival distributions were found among three different cohorts (both p < 0.001).

The survival probability at 1, 3 and 5 years were 91%, 67%, 47% for 2004–2007 cohort, 88%, 67%, 49% for 2008–2011 cohort, and 90%, 75%, 72% for 2012–2015 cohort, respectively, in patients within the Milan criteria. The survival probability at 1, 3 and 5 years were 53%, 29%, 18% for 2004–2007 cohort, 45%, 25%, 16% for 2008–2011 cohort, and 56%, 37%, 33% for 2012–2015 cohort, respectively, in patients beyond the Milan criteria. Significant survival distributions were found in three different periods of HCC patients for both groups (Fig. 4A,B; p < 0.001).

**Figure 5:**
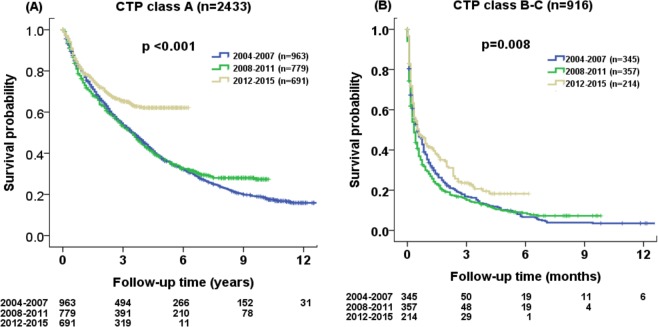
Comparison of survival distribution in three different time periods (2004–2015) according to (**A**) Child-Turcotte-Pugh (CTP) class A and (**B**) CTP class B or C. Significant differences in survival were found among three different cohorts in CTP class A (both p < 0.01).

The survival probability at 1, 3 and 5 years were 80%, 55%, 37% for 2004–2007 cohort, 76%, 54%, 38% for 2008–2011 cohort, and 81%, 65%, 62% for 2012–2015 cohort, respectively in patients with CTP class A. The survival probability at 1, 3 and 5 years were 38%, 17%, 10% for 2004–2007 cohort, 31%, 16%, 10% for 2008–2011 cohort, and 44%, 23%, 18% for 2012–2015 cohort, respectively, in patients with CTP class B or C. The 2012–2015 patient cohort had the best survival probability as compared with the 2004–2007 and 2008–2011 cohort for both CTP class A (p < 0.001; Fig. 5A) and class B-C patients (p = 0.008; Fig. 5B).

## Discussion

Our study shows that there was 13.1% reduction and 20.3% further reduction in the incident cases of HCC from 2004–2007 (n = 1308) to 2008–2011 (n = 1136), and from 2008–2011 to 2012–2015 (n = 905), respectively. HBV is one of the etiologies of liver cirrhosis and HCC. The incidence of HBV-related HCC declined in this 12-year study period. Consistent with previous cohort studies^6,8,9^, HBV infection significantly decreased after the implementation of the vaccination program^8,9^. Notably, the impact of national HBV vaccination program not only reduced the carrier rate of HBsAg, but also decreased the incidence of HBV-related HCC.

High levels of serum HBsAg and DNA are tightly associated with the occurrence of HCC^28–30^. Antiviral therapy using nucleoside or nucleotide analogues may inhibit HBV replication, and leads to improvement in liver histology and reduced risk of HCC^12,31^. In addition to HBV, HCV is also another risk factor for HCC globally^32^. Antiviral therapy for hepatitis C with interferon and ribavirin results in improved clinical outcomes by decreasing the risk of hepatic decompensation and HCC^33^. Alternatively, the development of cirrhosis is associated with non-alcoholic steatohepatitis (NASH). In addition, metabolic syndrome such as diabetes and obesity, could increase the risk of HCC in NASH patients^2,3^. In our study, the percentage of cryptogenic cause of HCC increased from 14% in 2004–2007 to 20% in 2012–2015; suggesting NASH may play an important role in inducing HCC.

The changing incidence of HCC in the international setting revealed that the incidence of HBV-related HCC has declined in most Asian countries such as China, Philippines and South Korea after the implementation of HBV vaccination. The decreasing rate of HCV-related HCC was reported in Japan and Italy due to specific antiviral therapy, including direct acting antiviral agents. The increasing rates of cryptogenic HCC were observed in US, Western countries and some Asian countries possibly due to the emergence of metabolic syndrome and non-alocholic steatohepatitis (NASH)^34^. These results are mostly consistent with our single center study in Taiwan.

The diagnosis of early stage HCC increased from 33% in 2004–2007 to 37% in 2012–2015 in this survey. The percentage of patients receiving curative treatment also increased from 44% in 2004–2007 to 55% in 2012–2015. Previous studies suggested that serum AFP and abdominal sonography were useful screening tools in high risk patients^2,35^. Our data were consistent with previous study^36^, indicating that screening patients with known risk factors may result in early cancer detection. Consistently^37,38^, our results also show that there is increased long-term survival in HCC patients because of a higher rate of curative treatments, including surgical resection and local ablation therapy.

The key prognostic predictors for HCC are liver functional reserve, tumor burden and therapeutic strategy. We further performed a subgroup analysis to delineate the pattern and cause of a better long-term survival in the 2012–2015 cohort. Importantly, we found that such survival impact is independent of CTP class, Milan criteria and treatment modality. An overall improvement in antiviral therapy for chronic viral hepatitis, cancer detection and active anti-cancer therapy over the study period may greatly contribute to the survival advantage^36,37^.

To date, several HCC staging systems have been implemented for prognostic prediction. However, the optimal staging system has been in intense debate for a decade. Our results suggest that the CLIP score was the best staging system for prognostic prediction in the cohort of 2004–2007 and 2008–2011. However, TIS system is a better system in discriminating clinical outcomes than the other 6 models for the 2012–2015 cohort. This discrepancy could be due to the change in patient demographics and pattern of treatment strategy, and also well explains why published studies addressing this issue revealed discordant results.

Our study has some limitations. First, this is a single center study in an area where HBV is commonly seen, which is different from Western countries. Second, selection bias could not be completely avoided because of the retrospective nature of this study. Third, a direct causal relationship between the changes of disease presentation and patient outcome cannot be confidently confirmed. Further studies are needed to validate our result.

In conclusion, the characteristics of patients with HCC have significantly changed over the last 12 years. HBV- and HCV-associated HCC became less common and cryptogenic HCC, probably related to NASH, was increasing. Early cancer detection and implementation of active anti-cancer treatment become possible and can be expected to prolong the long-term survival of HCC patients further.
