# Author Correction: Evolution of etiology, presentation, management and prognostic tool in hepatocellular carcinoma

**DOI:** 10.1038/s41598-020-75523-6

**Published:** 2020-10-23

**Authors:** Shu-Yein Ho, Po-Hong Liu, Chia-Yang Hsu, Cheng-Yuan Hsia, Yi-Hsiang Huang, Hao-Jan Lei, Chien-Wei Su, Rheun-Chuan Lee, Ming-Chih Hou, Teh-Ia Huo

**Affiliations:** 1 Department of Medicine, Taipei Veterans General Hospital, Taipei, Taiwan; 2 Department of Surgery, Taipei Veterans General Hospital, Taipei, Taiwan; 3 Department of Radiology, Taipei Veterans General Hospital, Taipei, Taiwan; 4 Department of Medical Research, Taipei Veterans General Hospital, Taipei, Taiwan; 5 Faculty of Medicine, National Yang-Ming University School of Medicine, Taipei, Taiwan; 6 Institute of Clinical Medicine, National Yang-Ming University School of Medicine, Taipei, Taiwan; 7 Institute of Pharmacology, National Yang-Ming University School of Medicine, Taipei, Taiwan; 8 Department of Internal Medicine, University of Texas Southwestern Medical Center, Dallas, Texas USA; 9 Division of Gastroenterology and Hepatology, University of Michigan, Ann Arbor, MI USA

Correction to: *Scientific Reports* https://doi.org/10.1038/s41598-020-61028-9, published online 03 March 2020

This article contains errors in Figure 3, where a typographical error occurred in the preparation of panels (a) and (b). The correct Figure 3 appears below as Figure 1.

**Figure 1:**
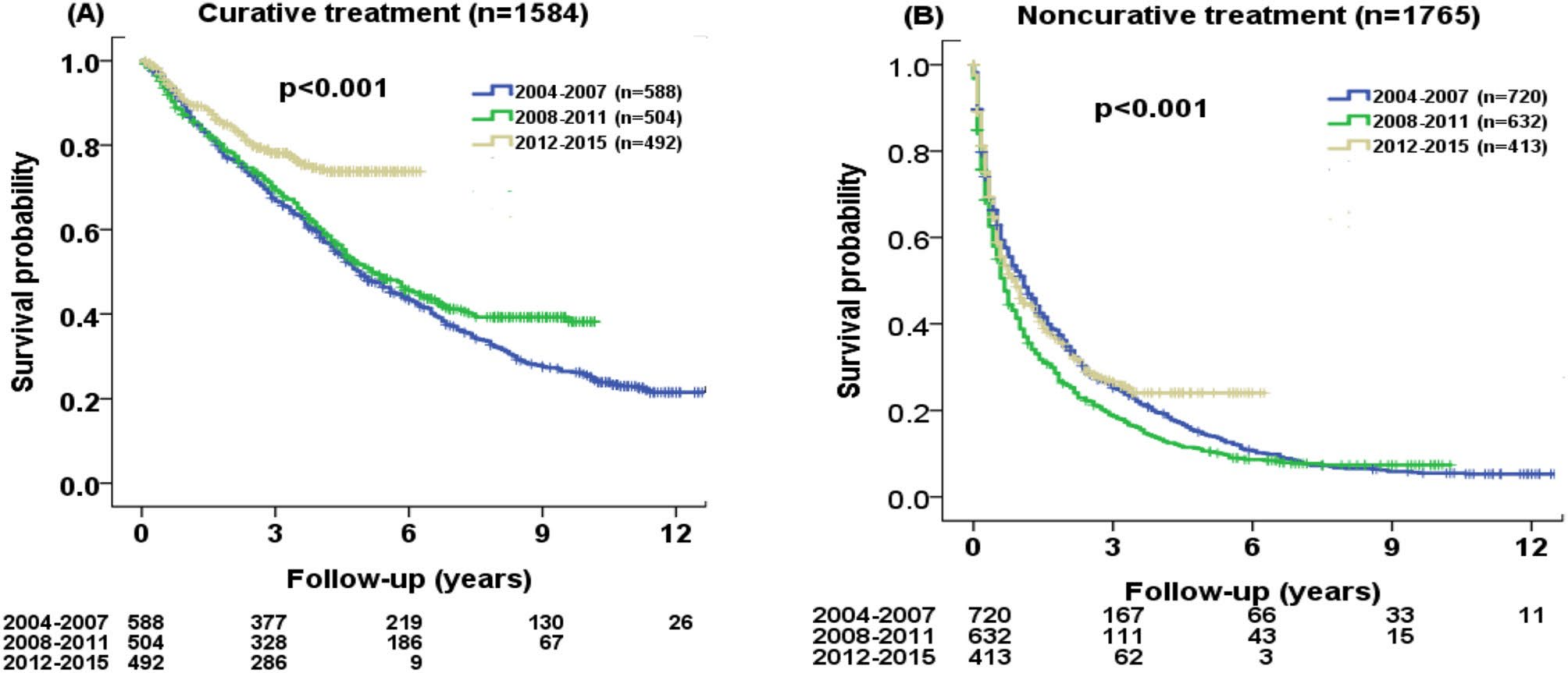
Comparison of survival distribution in three different time periods (2004–2015) according to (**A**) curative treatment and (**B**) non-curative treatment. Significant differences in survival distributions were found among three different cohorts (both p < 0.001).

